# Biomarkers of foraging and reproduction in captive adult female hawksbill sea turtles (*Eretmochelys imbricata*)

**DOI:** 10.1093/conphys/coag003

**Published:** 2026-01-28

**Authors:** Joslyn Blessing Kent, Kari Renee Dawson, Shingo Fukada, Masae Makabe, Isao Kawazu, Ken Maeda, Roldán A Valverde

**Affiliations:** 808 North Pine Street, Department of Biological Sciences, Southeastern Louisiana University, Hammond, LA 70402, USA; 808 North Pine Street, Department of Biological Sciences, Southeastern Louisiana University, Hammond, LA 70402, USA; Okinawa Churaumi Aquarium, Okinawa 905-0206, Japan; Okinawa Churaumi Aquarium, Okinawa 905-0206, Japan; Okinawa Churaumi Aquarium, Okinawa 905-0206, Japan; Okinawa Churashima Foundation Research Institute, 888 Ishikawa, Okinawa 905-0206, Japan; Okinawa Churaumi Aquarium, Okinawa 905-0206, Japan; One W. University Blvd, School of Earth, Environmental, and Marine Sciences, The University of Texas Rio Grande Valley, Brownsville, TX 78520, USA; 4581 NW 6th St Suite A, Sea Turtle Conservancy, Gainesville, FL 32609, USA

**Keywords:** Hawksbill sea turtle, β-hydroxybutyrate, sea turtle endocrinology, Captive held, Foraging physiology

## Abstract

Hawksbill sea turtles (*Eretmochelys imbricata)* are listed as critically endangered by the International Union for the Conservation of Nature (IUCN). To implement best conservation practices for this species, its biology should be well understood. Attempting to characterize the foraging physiology of free-ranging hawksbill sea turtles is complicated by the fact that sampling is typically limited to nesting females during the reproductive season. Without data from non-reproductive periods, it is difficult to determine whether observed physiological values reflect baseline conditions or are specific to the energetically demanding nesting season. Accordingly, in this study, we described the physiology of foraging in a captive-held population of hawksbill sea turtles for an entire year. Across the year, we sampled a total of five captive adult female hawksbills at the Okinawa Churaumi Aquarium in Okinawa, Japan. We measured the concentration of β-hydroxybutyrate (BHB), triglycerides (TRGs) and testosterone. Foraging biomarkers BHB and TRGs were both significantly higher during gonadal recrudescence and breeding than during gonadal quiescence, consistent with mature animals that were not foraging actively during breeding activities. Testosterone concentration also was higher during breeding months than during non-breeding months, especially in May, which marked the onset of mating. Elevated BHB during breeding activities indicated that captive hawksbills accumulated energy reserves during the non-breeding season to invest it in breeding activities. Additionally, elevated TRGs are correlated to vitellogenesis occurring in the breeding female hawksbills.

## Abbreviations

BHB, β-hydroxybutyrate;
TRG, triglycerides

## Introduction

Researching marine migratory vertebrates is a difficult task when considering the vastness of the ocean. Studying such organisms can be time consuming, costly and logistically difficult. Tracking, finding and recapturing the same migratory individual for multiple analyses is unlikely (Price *et al.*, 2012). Further, obtaining tissue samples, including blood, requires being close to an animal for a long enough period, which can be an operationally complicated process.

Free-ranging wild sea turtles are examples of species that are challenging to consistently sample across a year due to their expansive marine habitat and long migrations. Adult hawksbill sea turtles (*Eretmochelys imbricata*), like other sea turtle species, are characterized by long-distance migrations with strong homing abilities to return to their natal regions for reproduction (Velez-Zauzo *et al.*, 2008; Hawkes *et al.*, 2012; Esteban *et al.*, 2015). A record of an adult hawksbill turtle travelling as far as 705 km in 14 days for foraging and nesting activities (Hart *et al.*, 2012) illustrates sampling difficulty.

To understand seasonal trends in the physiology of sea turtles, year-round sampling is crucial. Many studies have focused on the biology of hatchlings and on the nesting ecology of adult female turtles due to the accessibility of the animals on beaches while hatching and nesting (Lutz *et al.*, 1996). Fewer studies have focused on free-ranging wild sea turtle biology when away from nesting beaches.

Utilizing captive animals is one way to study the same individuals over an entire reproductive cycle. However, even if a captive individual belongs to the same species as one observed in the wild, there may be physiological differences between the two. When discussing differences in captive and wild sea turtles specifically, one must consider that captive animals are fed regularly, lack predation pressure and do not migrate. Breeding programmes exist for captive sea turtles in which breeding and foraging conditions are largely controlled (Maggeni and Feeney, 2020). Captive studies can serve as a point of reference for wild population studies, but their validity can also be supported by wild populations’ studies (Price *et al.*, 2012). For example, captive Kemp’s ridley turtles (*Lepidochelys kempii*) kept under semi-natural conditions have been shown to display similar endocrine profiles to those of wild animals of the same species (Rostal, 2004).

Studies conducted on captive sea turtles are rare but contribute greatly to the foundation of sea turtle knowledge (Wood, 2022). For instance, juvenile captive green sea turtles (*Chelonia mydas*) exhibit an increased concentration of β-hydroxybutyrate (BHB) during fasting periods, supporting the observation that BHB is a ketone body produced by vertebrates via ketogenesis when exogenous glucose is restricted over a period of a few days (Price *et al.*, 2012; Jensen *et al.*, 2020). On the other hand, there is an elevation in triglyceride (TRG) concentration in captive juvenile green turtles during periods of feeding, attributed to macronutrients from ingested food being repackaged as TRG in the liver and subsequently transported to adipose tissue via the bloodstream (Price *et al.*, 2012). TRG concentration has also been described to increase during reproduction in birds and reptiles in response to vitellogenesis and folliculogenesis (Vanderkist *et al.*, 2000; Vezina and Williams, 2003; Gorman *et al.*, 2009; Price, 2017). Oestrogen induces vitellogenin (VTG) and very-low-density lipoprotein (VLDL) production from the liver, which carry phospholipids and TRG to support the development of oocytes in the ovary (Price, 2017). Thus, both VTG and TRG increase during folliculogenesis, and TRG also increases during periods of active foraging in reproductively quiescent sea turtles. Thus, TRG may be confounded if used as a marker of feeding during the reproductive season.

Testosterone dynamics have been documented in both wild and captive sea turtles. In wild populations of green, loggerhead and hawksbill sea turtles, testosterone has been observed to steadily decrease as the nesting season ensues and has been shown to reach a nadir by the end of the nesting season (Dobbs *et al.*, 2007; Smelker *et al.*, 2014; Bruno *et al.*, 2021). In adult female Kemp’s ridley sea turtles, testosterone followed the same pattern of peaking during the onset of mating and steadily decreased with each clutch oviposited in wild turtles as well as in those held in semi-natural, captive conditions (Hamann *et al.*, 2002; Rostal, 2004). Although the physiology may differ between captive and wild animals, testosterone trends in reproductively active female sea turtles have been observed to be consistent whether held captive or sampled in the wild.

Based on observations of BHB, TRG and testosterone in previous studies on sea turtles, these biomarkers of foraging and reproduction could be used to make predictions about breeding strategies. Income and capital breeding are two strategies that are often compared to each other while examining the relationship between allocation of energy and breeding functions (Jonsson, 1996; Bonnet *et al.*, 1998; Stephens *et al.*, 2009). Income breeders continuously forage during their reproductive season and use that energy to reproduce, whereas capital breeders fuel reproduction from energy that was stored prior to reproductive events (Jonsson, 1996; Bonnet *et al.*, 1998). Capital and income breeders are opposite ends of a continuum, where interpretation of these categorizations has led to some subjectivity (Stephens *et al.*, 2009; Kerby and Post, 2013). Identifying where on the spectrum a species most aligns can help characterize crucial feeding grounds. However, sea turtles do not appear to conform to these definitions since they forage actively during much of folliculogenesis. Nevertheless, energy acquisition and allocation are critical aspects of the reproductive process. Since foraging is related to reproductive success (Vollrath, 1987), the foraging grounds should be protected for endangered species like the hawksbill sea turtle (Bjorndal, 1999).

The aim of this study was to analyse the concentrations of BHB, TRG and testosterone over an entire year in captive adult female hawksbill turtles. The information generated will serve as a reference for wild hawksbill populations and facilitate the elucidation of whether sea turtles forage actively during their breeding season. The data and conclusions will help fill gaps in physiological data unavailable in animals pre- and post-nesting season.

## Materials and Methods

### Sampling collection

All sea turtle handling protocols were approved by Southeastern Louisiana University’s Institutional Animal Care and Use Committee (IACUC) permit number 0086. Plasma from five adult female hawksbills was collected between April 2018 and March 2019 in Okinawa Churaumi Aquarium in Okinawa, Japan. Nesting season for this species in the northern hemisphere is April–August, and the non-nesting season is considered as September–March. In captive sea turtles, breeding activities have included mating and nesting (Licht *et al.*, 1979; Wood and Wood, 1980), whereas in wild populations, these included only mating, with nesting season as a separate period (Hays *et al.*, 2010). Here, we added to those reproductive activities those associated with follicular development (ovarian recrudescence) and provided ovarian ultrasonograms. Three of the individuals were sampled every 2 weeks during 22 April 2018 to 21 March 2019, and every day during 1 week of mating in mid-May and 1 week of nesting in mid-June. Two of the individuals were sampled every 2 weeks during April 2018 to May 2018, and every day for a week during mating in May of the same year.

**Figure 1:**
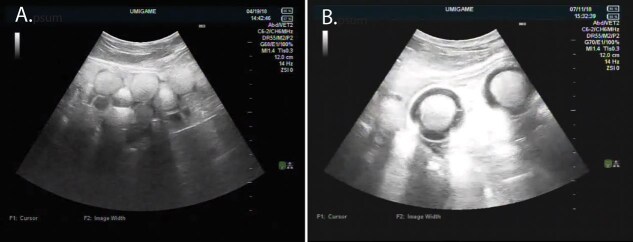
Representative ultrasound scans of captive-kept hawksbill sea turtles included in this study. (a) Ultrasound scan conducted in April of 2018 showing developed ovarian follicles. (b) Ultrasound scan conducted in July of 2018 showing calcified eggs in oviduct.

Female turtles were placed individually in a tank with a male for 1 week to allow mating. Follicular development occurred during April as determined by ultrasound scans of the ovaries, and mating behaviour occurred in May for all turtles; eggs were observed in July (Fig. 1). Egg laying events were monitored and documented. Since turtles were mated at contrasting times, the timing of reproductive events was not identical for each turtle. Additionally, qualitative observations were made regarding the turtles’ feeding status over the course of sampling and were categorized by eating well, partially or not at all. If a turtle was categorized as ‘eating well’, food intake was approximately 2% of their body weight.

The straight carapace length of the turtles in this study ranged from 75.5 to 84 cm. The body weight of the animals ranged from 53 to 82.2 kg, with the average weight being 62.5 kg. Animals were mated in an indoor tank with dimensions 6.25 × 2.5 × 2.5 m^2^ and water depth of 1 m. The dimensions of the outdoor rearing/egg laying pool were 176.4 × 16.8 × 10.5 m^2^ and had a water depth of 2 m. Water intake came from 200 m offshore and was filter circulated for the rearing/egg laying pool. The average water temperature for the year was 24.9°C, with the minimum being in February at 22.1°C and a maximum of 28.8°C in August. Eggs were laid in a 115-m^2^ outdoor sandy nesting area. The turtles were generally fed five times per week. Their diet mainly consisted of frozen banded blue sprat (*Spratelloides gracilis*), capelin (*Mallotus villosus*) and squid with fresh fish (such as tuna) occasionally provided. Additionally, vitamin supplements for aquatic animals were added.

Circulating TRG concentration was analysed in 88 samples collected from the five different turtles during April 2018 to March 2019. TRG concentration was measured on site with a Fuji DRY-CHEM 7000 V biochemistry autoanalyser (FUJIFILM Corporation, Japan) for animals, as previously described (Kawazu *et al.*, 2015). The analyser’s measurement of TRG occurs by hydrolysis and then measurement of glycerol; it therefore includes pre-existing free glycerol in plasma. The inter-assay coefficient of variation (CV) for the TRG assay was 4.75% and the intra-assay CV was 0.5%.

Circulating BHB concentration was measured in 120 samples from the five different turtles collected during April 2018 to March 2019. To measure BHB concentration, we used a commercially available colorimetric assay (Cayman Chemical, Item No. 700190), following manufacturer’s specifications. Assay plates were read at 450 nm. For this, we first diluted the samples 10-fold and ran in duplicate. BHB concentrations were generated using a regression equation obtained from their standard curves. Triplicates of an individual sample were run within and between BHB assays for quality control and to calculate intra- and inter-assay variability. The inter-assay CV for BHB assays was 4.1%, and the intra-assay CV was 8.9%.

Circulating testosterone concentration was measured in 119 samples from the five different turtles collected during April 2018 to March 2019. To measure testosterone concentration, we conducted a steroid extraction of 25 μl of plasma and added 175 μl of testosterone assay buffer to bring all samples to 200 μl. We then added 2 ml of diethyl ether and placed the mix into a slurry of 80% ethanol and dry ice to freeze the aqueous phase. We then decanted the ether phase into a test tube containing 1 ml of ddH_2_O to eliminate impurities. The freeze-decant step was repeated into a clean test tube. We evaporated the ether under a stream of nitrogen gas while in a warm water bath (37°C) and reconstituted with 250 μl of testosterone assay buffer. We analysed the samples using an Enzyme-linked Immunosorbent Assay (ELISA) Kit (ENZO Life Sciences, Cat. No. ADI-900-065) following the manufacturer’s specifications and read at 405 nm. We determined testosterone concentration using the four-parameter logistic regression in SigmaPlot v14.0. A pool of serum from male and female loggerhead sea turtles captured across a year was spiked with a known concentration of steroid and was included in testosterone assays for quality control and to calculate intra- and inter-assay variability. The inter-assay CV for the testosterone assay was 6.8%, and the intra-assay CV was 9.5%.

The statistical analysis for the data was done using R Studio. Normality of the data was evaluated by conducting Shapiro–Wilk tests. The data were not significantly different than normal if the *P* value on the Shapiro–Wilk test was over 0.05. If the predictor *P* values were less than 0.05, the data were log-transformed for subsequent analysis. If the data were not normally distributed after a log transformation, a Kruskal–Wallis rank sum test was performed.

## Results

**Figure 2:**
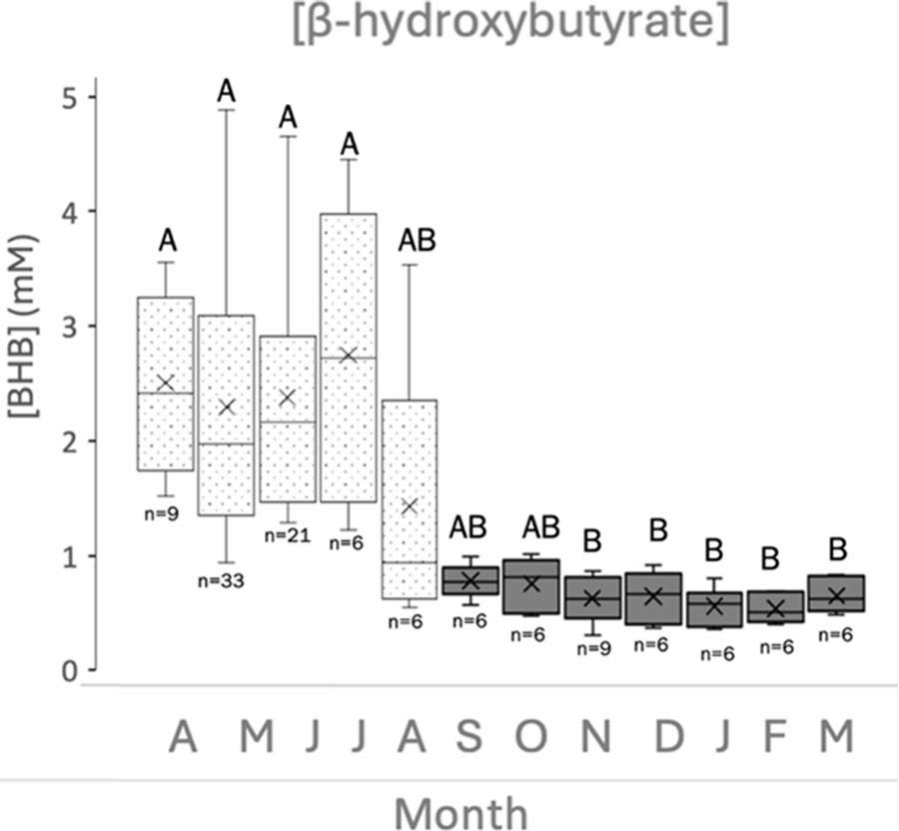
Mean concentration of BHB for each month, where *n* represents the number of samples collected per month. Dotted boxes represent months during the breeding season, and grey boxes represent non-breeding months. Boxes that share letters do not differ significantly from each other (*P* < 0.05). Whiskers indicate range; box represents inter-quartile range (percentile: 25–75%) with median.

For each of the three turtles that nested, there were six egg laying events, occurring in June, July and August. Analysis of BHB concentration showed that data were not normally distributed (*P* < 0.05). The data were thus log-transformed but did not correct for normality. Therefore, we used Kruskal–Wallis rank sum for subsequent analysis. BHB was significantly higher during the breeding season in April–July (*P* < 0.05) than in November–May (Fig. 2). The average BHB concentration for April, May, June and July was 2.51 (±0.3), 2.30 (±0.2), 2.38(±0.2) and 2.75 (±0.5) mM, respectively. February had the lowest average BHB concentration at 0.54 (±0.05) mM.

**Figure 3:**
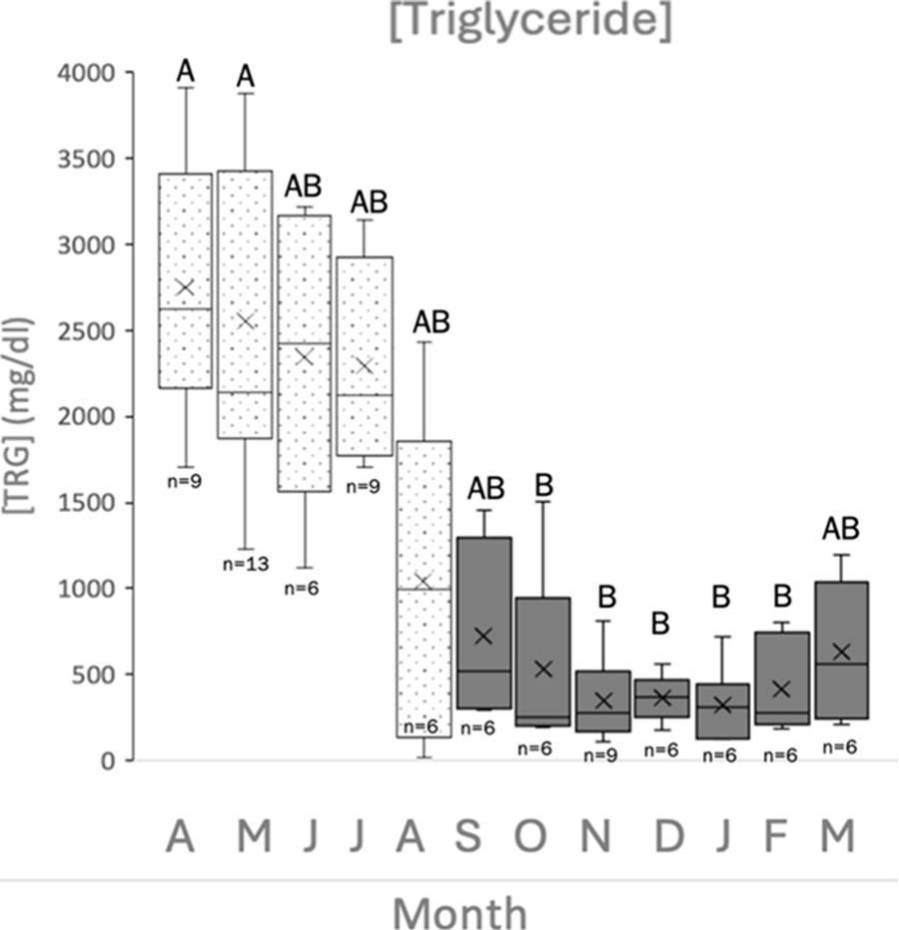
Concentration of TRG for each month, where *n* represents the number of samples collected per month. Dotted boxes represent months during the breeding season, and grey boxes represent non-breeding months. Boxes that share letters do not differ significantly from each other (*P* < 0.05). Whiskers indicate range; box represents inter-quartile range (percentile: 25–75%) with median.

Analysis of TRG concentration showed that the data were not normally distributed (*P* ≤ 0.05). TRG data were not normally distributed after a log transformation either (*P* ≤ 0.05). Therefore, a Kruskal–Wallis test was utilized to test for differences. Analysis showed that TRG concentration was highest during the onset of the breeding season in April–May, decreased across the season and significantly decreased in October (*P* ≤ 0.05) (Fig. 3). The average TRG concentration during April and May was 2748.22 (±245.8) mg/dl and 2552.92 (±252.2) mg/dl, respectively. January represented the lowest average TRG concentration at 325.50 (±88.3) mg/dl.

**Figure 4:**
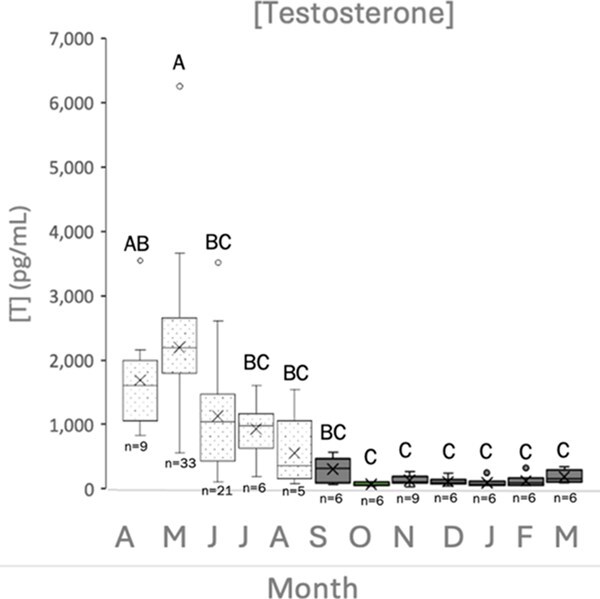
Mean T concentration for each month, where *n* represents the number of samples collected per month. Dotted boxes represent months during the breeding season, and grey boxes represent non-breeding months. Boxes that share letters do not differ significantly from each other (*P* < 0.05). Whiskers indicate range; box represents inter-quartile range (percentile: 25–75%) with median.

Analysis of testosterone concentration showed that data were not normally distributed (*P* ≤ 0.05). The data were then log-transformed but did not correct for normality, and therefore, we used Kruskal–Wallis rank sum for subsequent analysis. Testosterone was significantly elevated during breeding season, particularly during May (*P* ≤ 0.05) (Fig. 4). During May, the average testosterone concentration was 2196.20 (±191.0) pg/ml. The month of October showed the lowest average testosterone concentration at 70.46 (±9.4) pg/ml.

## Discussion

Reproductive and foraging functions are crucial components of the life history of sea turtles. Sea turtles are thought to be capital breeders, in which animals accumulate nutrients in body tissues to later support reproductive activities. However, because sea turtles forage during much of folliculogenesis, we prefer not to use this term, as well as its associated income breeding terminology. What has been established is that during ovarian recrudescence, sea turtles exhibit a period of aphagia (Owens, 1980), which ensues sometime before the onset of mating activities. It has been shown experimentally that aphagic juvenile green sea turtles exhibit an increase in circulating concentration of BHB, along with a decrease in TRG (Price *et al.*, 2012). The increase in BHB in aphagic sea turtles is a consequence of stored fat mobilization and subsequent ketone production (Price *et al.*, 2012). Reciprocally, circulating TRG increases after foraging resumes in juvenile green turtles (Price *et al.*, 2012). In the current study, we aimed to establish whether hawksbill sea turtles comply with the observation of fasting during breeding activities by sampling adult captive-held hawksbills across the year.

In the captive hawksbill turtles, BHB and TRG concentrations were both significantly higher during the breeding season than the rest of the year when the animals were not reproductively active. Elevated BHB during the month leading up to reproduction and decreasing as soon as the reproductive season was over is consistent with an animal experiencing aphagia during their breeding season (Price *et al.*, 2012). Accordingly, our data indicate that captive hawksbill sea turtles do not forage during breeding season. This is consistent with the qualitative observations that noted the turtles were either not eating or eating less during the months when BHB and testosterone were elevated compared to when BHB concentration was relatively low (data not shown). Further, Kawazu *et al.* (2015) observed that the inactive feeding ratio (a quantitative ratio calculated by comparing the number of days a turtle did not eat to the rest of the days in the month) of reproductively active captive-held hawksbill sea turtles was much higher during the reproductive season compared to months where there was less follicular development. During times of greatest follicular development, hawksbills were observed to have the most reduced appetite (Kawazu *et al.*, 2015). Green and hawksbill sea turtles in additional studies have been noted to exhibit a decreased appetite during reproduction (Bjorndal *et al.*, 1985; Goldberg *et al.*, 2013).

It has been hypothesized that the reason for the lack of appetite during reproduction could be attributed to vitellogenesis, since feeding is inhibited by oestrogen administration (Owens, 1980). Vitellogenesis is the deposition of yolk precursors, such as VLDLs and VTG, into the follicles in the ovary (Price, 2017). Vitellogenesis is stimulated by ovarian oestrogen (Owens, 1976), and therefore, this network of hormones and proteins are thought to play a role in suppressing appetite. This relationship is not fully understood and should be further investigated. Alternatively, the lack of appetite may be due to decreased abdominal space to ingest food since follicles are increasing in size leading to reproduction, which in turn limits the amount of food intake (Kawazu *et al.*, 2015).

Elevated TRG concentrations have been observed in reptiles and birds during their reproductive season, specifically during vitellogenesis (Price, 2017). During vitellogenesis, oestrogen secreted from follicular cells stimulates the secretion of VTG and VLDL, which travel to developing oocytes via the bloodstream (Blanvillain *et al.*, 2011; Kawazu *et al.*, 2015; Price, 2017). Lipoproteins VTG and VLDLs are both yolk precursors, which are responsible for transporting phospholipids and TRG, respectively (Price, 2017). In reptiles, such as sea turtles, during periods where VLDL concentration is high, TRG concentration should be expected to be high as well. This trend has been observed in yolk deposition in green sea turtles (Hamann *et al.*, 2002), in hawksbill sea turtles (Goldberg *et al.*, 2013), and has been reviewed (Price, 2017).

In the present study, TRG exhibited a gradual decreasing trend over the course of reproductive activities in our captive hawksbills, remaining at low concentration in the off-season. This decrease paralleled the concentration profile exhibited by VTG, because both VTG and VLDL are produced by oestrogen stimulation, which wanes at the end of the reproductive season.

In juvenile green sea turtles, TRG exhibited low concentration and high BHB concentration in times of fasting, and the same turtles in a fed state exhibited elevated TRG and low BHB concentrations (Price *et al.*, 2012). Individuals in that study were reported to be between 19 and 20 kg, which are consistent with juvenile weights for this species (Seminoff *et al.*, 2015). The results from our study oppose these observations, since both BHB and TRG followed the same trend over a year. Given that Price *et al.* (2012) studied juveniles, vitellogenesis and follicular development would not yet be occurring. Since vitellogenesis was occurring in the reproductively active female hawksbill sea turtles in our study, the TRG assay detected much higher amounts due to elevated lipoprotein during breeding activity. Thus, TRG may be a more appropriate biomarker of food intake when animals are not reproductively active and follicular development is not occurring.

Kawazu *et al.* (2015) sampled two captive-kept hawksbill turtles during 2004–2009. During the first year, there was no follicular development, and TRG concentration did not exceed 885.7 mg/dl and showed no seasonal fluctuations. From 2006 through 2009, when follicular development was occurring, TRG peaked between 2657.1 and 3542.8 mg/dl during the onset of the reproductive season and gradually decreased the remainder of the year (Kawazu *et al.*, 2015). In captive green sea turtles, TRG concentration was noted to peak during courtship and early in the nesting season, and to have the lowest concentrations in atretic and non-vitellogenic females (Hamann *et al.*, 2002). These patterns are consistent with the trends and values observed in the hawksbill sea turtles sampled in the current study.

Behavioural and physiological observations allowed us to discern reproductive behaviours and processes in this study, including follicular development (occurring in April), mating (occurring in May) and egg laying (occurring June–August). While many studies define breeding season as the time spent mating and nesting, we have included follicular development as it represents the physiological onset of reproduction, marked by increased reproductive hormones and oocyte development (Rostal *et al.*, 1997; Pérez-Bermúdez *et al.*, 2012). Additionally, to ascertain the periods of reproductive versus non-reproductive activity, we measured testosterone. Because testosterone is produced by developing and mature ovarian follicles, this biomarker of reproduction is elevated during pre-breeding and breeding activities (Rostal *et al.*, 1997, 1998). We observed that testosterone was higher in reproductive months when compared to non-reproductive months, especially in May, which marked the onset of mating. This suggests that our captive animals were exhibiting typical reproductive physiology of wild, free-ranging animals. A similar observation was made about the reproductive physiology of captive and free-ranging Kemp’s ridley sea turtles (Rostal *et al.*, 1998), suggesting that the comparisons we have made in the present study are appropriate. In this study, testosterone exhibited a clear decrease across the nesting season, which has been observed in hawksbills, as well as in other species including green, loggerhead and Kemp’s ridley sea turtles (Hamann *et al.*, 2002; Dobbs *et al.*, 2007; Smelker *et al.*, 2014; Bruno *et al.*, 2021).

Overall, our BHB data indicate that hawksbills did not forage during breeding activity, assuming that our animals behaved naturally. TRG and testosterone were elevated early in the reproductive months and decreased through the season, matching previous studies. The results we obtained regarding the physiology of our captive animals support the natural behaviour of our animals. The findings presented in this study can help develop effective breeding programmes and assist conservation efforts. Additionally, future work should be conducted to validate the comparisons made between captive and free-ranging hawksbill sea turtles. More specifically, research should be directed to investigating the concentrations of metabolites during the non-nesting season of free-ranging turtles. This can help inform conservation and foraging ground management strategies.

## Data Availability

The data underlying this article will be shared on reasonable request to the corresponding author.
